# Zero Discharge of Dyes and Regeneration of a Washing Solution in Membrane-Based Dye Removal by Cold Plasma Treatment

**DOI:** 10.3390/membranes12060546

**Published:** 2022-05-25

**Authors:** Hee-Jun Kim, Uje Lee, Hyun-Woo Kim, Min Cho, Jaewoo Lee

**Affiliations:** 1 Department of Polymer-Nano Science and Technology, Jeonbuk National University, 567 Baekje-daero, Deokjin-gu, Jeonju-si 54896, Republic of Korea; rlagml3377@jbnu.ac.kr (H.-J.K.); luj9880@jbnu.ac.kr (U.L.); 2 Jeonbuk Green Environment Center, 567 Baekje-daero, Deokjin-gu, Jeonju-si 54896, Republic of Korea; 3 Department of Environmental Engineering, Jeonbuk National University, 567 Baekje-daero, Deokjin-gu, Jeonju-si 54896, Republic of Korea; hyunwoo@jbnu.ac.kr; 4 Division of Biotechnology, Advanced Institute of Environment and Bioscience, College of Environmental and Bioresource Sciences, Jeonbuk National University, Iksan 54596, Republic of Korea; 5 Department of Bionanotechnology and Bioconvergence Engineering, Jeonbuk National University, 567 Baekje-daero, Deokjin-gu, Jeonju-si 54896, Republic of Korea

**Keywords:** dye removal, membrane adsorption, zero discharge, cold plasma treatment, membrane regeneration, washing solution regeneration

## Abstract

Although dye removal from wastewater streams has been investigated via several approaches using adsorbents, resins, or membranes, it is still hard to avoid the fact that dyes are persistently left in the adsorption materials or washing solutions used to regenerate the used adsorbents. In particular, given that cleaning agents are composed of acid/base, organic solvents, or electrolytes, dye adsorption and adsorbent regeneration processes leave behind more hard-to-manage wastewater containing dyes. In this study, we demonstrated that cold plasma (CP) treatment, which is one of the advanced oxidation processes (AOPs), can be used for zero discharge of dyes and regeneration of a washing solution in a membrane-based dye removal process. Specifically, CP treatment was found to successfully remove dyes released from a washing process to regenerate a used membrane, thereby effectively recycling a cleaning solution. As a result, the regenerated washing solution was more favorable for the adsorbed dyes’ elution, leading to the successful regeneration of a used membrane without a significant loss of dye removal efficiency. This fact was evidenced by a comparative study on the effect of CP treatment on the reusability of membranes and washing solutions and the kinetic analysis of the AOP of the desorbed dyes. We hope that this study contributes to opening a new door for environmentally friendly and sustainable dye removal.

## 1. Introduction

Today, one-third of the world’s population lives in water-deficient countries [1]. Furthermore, the water shortage problem has been accelerated by hard-to-degrade pollutants released from various anthropogenic activities, ranging from the semiconductor industry to the textile industry [2]. Accordingly, hard-to-handle contaminants should be addressed to make water reuse readily available, thereby alleviating water stress. However, we still have a long way to go to ensure complete control of persistent pollutants. For example, tetramethylammonium hydroxide (TMAH, C_4_H_13_NO), which is used as an etching agent in the photolithography process of the semiconductor industry, is considered difficult to eliminate by secondary (i.e., biological) wastewater treatment in that it is highly likely to inactivate microorganisms in the activated sludge used for biological wastewater treatment [3]. When it comes to the pollutants involved in the wastewater discharged from pharmaceutical, cosmetic, edible oil, paint, fur, leather, paper, and textile manufacturing industries [4,5,6,7,8,9], it is not too much to say that dyes could be second to none from the perspective of priority for removal from wastewater considering that they pose significant harm to humans.

Methylene blue, which is a cationic organic dye commonly found in textile wastewater [10], is a typical example that reveals the harmfulness of dyes. For instance, methylene blue is so toxic and harmful to humans that it can cause nausea, anemia, diarrhea, and high blood pressure [11,12]. This is also the case for aquatic animals and plants [13]. Accordingly, upon consideration of environmental protection, human health, and sustainable development, dyes have to be adequately removed and must not be released into the aquatic environment. Unfortunately, however, dyes are also recalcitrant materials in biological wastewater treatment [14,15]. For that reason, people have tried to eliminate methylene blue from wastewater via diverse approaches, such as membrane filtration, adsorption, and oxidation processes [16]. Among the available technologies for dye removal, membrane separation has attracted much attention as a potential remedy for dye removal owing to the many favorable features it offers: high quality of processed water, controllability of solids and hydraulic retention time, and reduction in excess sludge [17,18,19,20,21,22].

However, it does not necessarily mean that membrane separation always benefits dye removal. In short, membrane-based dye removal also has its own pros and cons. To be specific, dyes are typically removed by membrane separation via Donnan exclusion, size exclusion, and adsorption [2]. Here, since adsorption contaminates the membrane surface and sublayer with dyes, a washing process is necessary to regenerate used membranes for reuse. However, even though a used membrane is recycled after washing, desorbed dyes from a membrane are left persistently in the washing solution after the cleaning process. Even worse, given that an aqueous washing solution usually contains ethanol, acetone, hydrogen chloride, sodium hydroxide, or sodium chloride [4,23,24,25,26,27,28], a used washing solution including desorbed dyes could provide a source of hard-to-manage secondary contamination.

**Figure 1:**
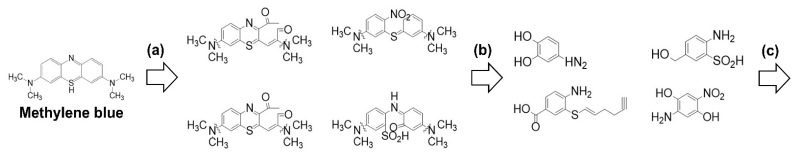
Illustration of degradation of methylene blue by reactive oxidizing species generated by a CP system [35,36]. (**a**) Generated radicals attack methylene blue. (**b**) Further oxidation of reaction intermediates. (**c**) Mineralization producing NO_3_^−^, SO_3_^−^, CO_2_, H_2_O, and so on.

With this in mind, we investigated the possibility of zero discharge of dyes and regeneration of a washing solution by degrading desorbed dyes in a washing solution using an advanced oxidation process (AOP). Among several AOPs, such as Fenton-, UV-, ozone-, and cold plasma (CP)-based AOPs, CP treatment is known to offer strengths such as higher oxidation potential, lower energy consumption, and shorter treatment time at room temperature and atmospheric pressure independently of the content of organic foulants, pH, and turbidity, unlike other AOPs [29,30,31]. The higher oxidation potential stems from reactive oxidizing species, including several radicals, such as O_2_·, OH·, HO_2_·, or their recombination [15,32], capable of non-selectively oxidizing various materials [33,34], which is also expected to be effective in degrading methylene blue, as shown in Figure 1. Moreover, CP treatment can be applied to the existing infrastructure without installing additional reactors. Therefore, all things considered, we chose CP treatment and tried to demonstrate its feasibility for zero discharge of dyes and regeneration of a washing solution.

## 2. Materials and Methods

All chemicals used for this study were used without further purification. Ultrafiltration (UF) membranes were fabricated with a polymer solution consisting of 20 wt% polysulfone (PSf; UDEL^®^ P-3500 LCD MB7, Solvay Korea, Seoul, Korea) and 80 wt% N-methyl-2-pyrrolidinone (NMP; Samchun, Seoul, Korea) via non-solvent-induced phase separation (NIPS) as reported previously [37]. Specifically, the polymer solution was poured onto a solvent-soaked non-woven fabric and coated with a bird film applicator (K3540, Elcometer, Manchester, UK) with the coating thickness set to 200 μm. The coated polymer solution was immediately immersed in tap water and kept overnight for complete liquid–liquid demixing. The surface image of the as-prepared UF membrane was observed using a field emission scanning electron microscope (FESEM; Gemini 500, Carl Zeiss, Oberkochen, Germany) installed in the Center for University-Wide Research Facilities (CURF) at Jeonbuk National University after being sputter-coated with platinum.

**Figure 2:**
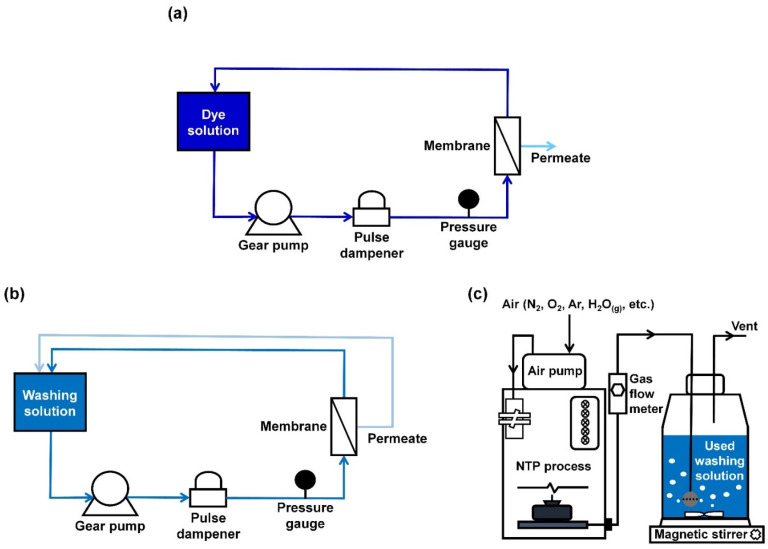
(**a**) Schematic illustration of the crossflow UF system to evaluate the pure water flux and dye removal performances of the as-prepared membrane. (**b**) Schematic illustration of the crossflow system configuration used to wash the used membrane. (**c**) Schematic illustration of a lab-scale set-up of CP treatment system.

The pure water flux (L m^−2^ h^−1^, LMH) and dye removal performances of the as-prepared UF membranes were evaluated with a lab-scale crossflow UF system, comprising a gear pump (EMG-4000, SCIST, Suwon, Korea), a pulse dampener, a pressure gauge, a flat-sheet membrane module, and a 2 L feed tank with a working volume of 1 L (Figure 2a). The effective membrane area, operating pressure, and crossflow rate were 13.75 cm^2^, 1.5 bar, and 1.8–2.1 L min^−1^, respectively. The permeate solutions were collected for 30 min to calculate fluxes. An average 9.7 ppm aqueous dye (methylene blue, Sigma-Aldrich, St. Louis, MO, USA) solution was used to assess the dye removal efficiency of the as-prepared UF membranes and intentionally stain a membrane. The dye concentrations of feed (*C_f_*) and permeate (*C_p_*) solutions were measured by means of ultraviolet–visible (UV–vis) spectroscopy (SP-UV1100, DLAB, China), and they were used to calculate the dye removal rate on the basis of the following relationship: (1 − *C_p_*/*C_f_*) × 100%.

After membrane filtration for dye removal, a dye solution was drained from the crossflow system, and then the used membrane was washed with a 50% aqueous alcohol (Samchun, Korea) solution at the same operating pressure and crossflow rate for 20 min. While a retentate solution was circulated in the crossflow system, a permeate solution was also returned to the washing solution tank during the cleaning process, as described in Figure 2b, upon consideration that a permeate solution is also supposed to contain a certain amount of dye since the dye removal rate is not 100%. After the washing process, the used washing solution was drained from the crossflow system and collected with desorbed dyes from a membrane for reuse, followed by a 10 min rinsing of the system with DI water three times. A series of filtration steps described above was repeated three times. In the meantime, the installed membrane was used repeatedly without disassembling a membrane module (i.e., clean-in-place) in order to determine whether CP treatment effectively degrades desorbed dyes in a washing solution and thereby improves the reusability of the used membrane and washing solution.

The CP treatment of a used washing solution was carried out with a customized lab-scale CP system (Groon Co., Ltd., Jeonju, Korea) between the previous clean-in-place step and following membrane filtration step for the purpose of removing desorbed dyes in the used washing solution prior to the reuse of the washing solution. The lab-scale CP system featured a threshold current of 10 mA and maximum output power of 2.2 W and consisted of an air pump (Shinhwahightech, ZP-25, Chungju, Korea), a CP unit equipped with an electrical circuit and an AC-DC converter (~10 kV), a gas flow meter, and a glass reactor (Figure 2c) [38]. While the ambient air passed through the CP unit at 5 L min^−1^, the supplied air was converted into reactive oxygen/nitrogen species and their derivatives and immediately sent to the reactor. The reactive species were used for the AOP of desorbed dyes in the used washing solution, and the removal efficiencies were monitored at regular intervals. On the basis of the obtained removal efficiencies, the kinetic study was carried out using SigmaPlot 14.0 (Systat Software, Inc., San Jose, CA, USA). In the exponential rise to maximum, the kinetic rate constant (*k*) was regressed on the basis of the following relationship: y = a (1 − e^(−*k*·x)^), where y is the removal efficiency (%) and x is the CP treatment time (min). Lastly, the energy per order (EEO) of each CP treatment was calculated using the following Equation [39]:

(1) $$\mathrm{EEO}=\frac{P\cdot t\cdot 1000}{V\cdot 60\cdot \log\left(\frac{C_{0}}{C_{t}}\right)}$$

where P is the power of the CP system (kW), t is the CP treatment time (min), V is the volume of dye solution (L), C_0_ is the initial dye concentration (ml L^−1^), and C_t_ is the dye concentration at time t (min).

## 3. Results and Discussion

**Figure 3:**
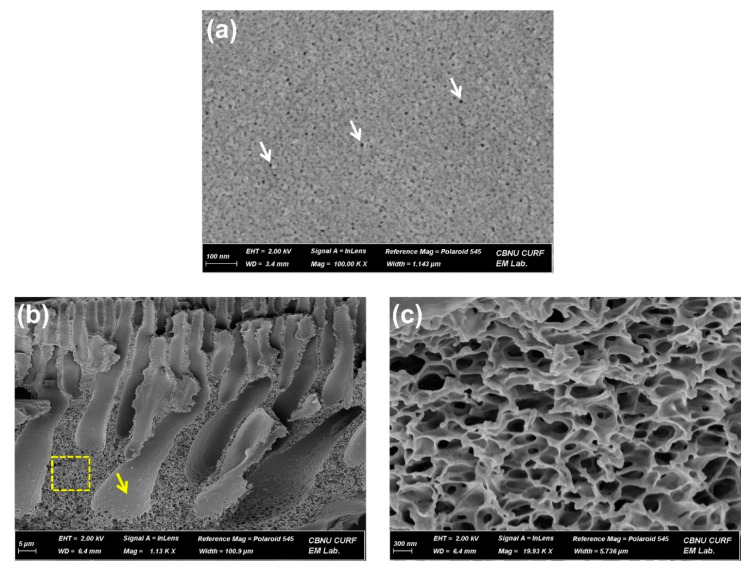
(**a**) Surface and (**b**) cross-sectional SEM image of the UF membrane prepared with a 20 wt% PSf solution. The white arrows indicate surface pores ranging from about 5 to 10 nm. The yellow arrow and dotted box mean finger-like and sponge-like regions, respectively. (**c**) The magnified SEM image of a sponge-like region.

**Figure 4:**
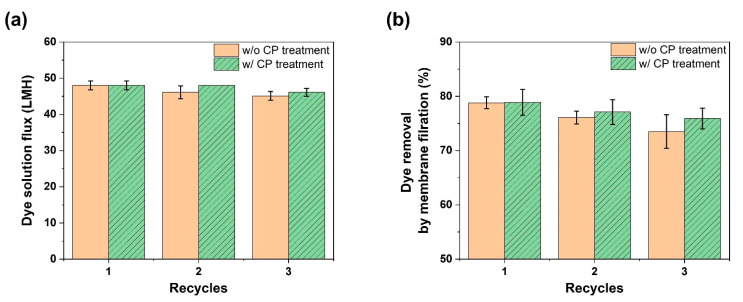
(**a**) Dye solution flux and (**b**) dye removal efficiency by membrane filtration (*n* = 3).

Figure 3a shows the surface SEM image of the as-prepared UF membrane with the surface pores ranging from 5 to 10 nm, while Figure 3b,c display the cross-sectional SEM images. As shown in Figure 3a, the UF membrane was well-fabricated, without any significant defects on the surface. The integral UF membrane prepared with a 20 wt% PSf solution exhibited 31.2 LMH bar^−1^ at 1.5 bar and about 2 L min^−1^ (Table 1), corresponding to a reasonable level as compared to the water permeability coefficient and dye removal rate of other UF membranes reported in the literature [40,41,42,43,44]. Subsequently, this membrane was used for dye removal under the same conditions as the above-mentioned one after 30-min membrane compaction using DI water. The as-prepared UF membrane revealed 25.5 LMH bar^−1^ and 78.8% removal in the first UF test using a 9.7 ppm dye solution in triplicate (Figure 4a,b). Afterward, the used membrane was thoroughly washed with a 50% aqueous alcohol solution for 20 min and was then rinsed using DI water for 10 min three times. The washing step using ethanol was expected to successfully detach the dye molecules physically adsorbed on membrane matrices of the surface and sublayer on the basis of the “like dissolves like” principle. Specifically, ethanol is one of the solvents with high solubility of methylene blue. Accordingly, methylene blue can be easily detached from membrane matrices by ethanol [45], enabling ethanol to regenerate the used membrane.

After the washing and rinsing procedures, the second UF test for dye removal was performed using the used membrane and newly prepared 9.7 ppm dye solution. The second UF test demonstrated that the dye removal rate was reduced from 78.8% to 76.1%, while the dye solution flux was maintained at a similar level (25.5 and 24.6 LMH bar^−1^ for the first and second UF tests, respectively) (Figure 4a,b). This reduction in the dye removal could be attributable to the incomplete washing and elution, leading to the loss of adsorption sites occupied by undetached dyes during the first washing process. By contrast, the flux was assumed not to be changed significantly, although dyes were adsorbed on a membrane, since the dye adsorption would dominantly occur on a membrane matrix on the surface along with finger-like region walls and sponge-like regions in the sublayer rather than the rims of surface pores. Accordingly, it was presumed that there was no significant change in the surface pore size after the dye adsorption. After the second UF test, the used membrane was washed with the used cleaning solution containing desorbed dyes, and the regenerated membrane was utilized for the third UF test. Likewise, the decrease (76.1% to 73.5%) in the dye removal rate appeared again in the third UF test (Figure 4a,b), whereas the dye solution flux represented only a marginal reduction (24.6 to 23.9 LMH bar^−1^).

On the other hand, the washing solution’s recycling accompanied by CP treatment was found to diminish the reduction in the dye removal rate. In detail, as the UF test for dye removal was conducted in triplicate, going through the same washing procedure intervened with the used cleaning solution’s oxidation by CP treatment, the dye removal rate gradually decreased from 78.9% to 75.9% while also keeping a similar dye solution flux within an acceptable deviation range (3.0%p) (Figure 4a,b). The gradual reduction in the dye removal rate implies that CP treatment induced a positive effect on dealing with the desorbed dyes in the used washing solution now that CP treatment would not enhance the washing capability of a 50% aqueous alcohol solution. Rather, one needs to recognize that the gradual drop in the dye removal rate was achieved even though the washing solution’s ability would have dwindled because the alcohol in a washing solution was also able to be oxidized and thereby partially removed by CP treatment.

**Figure 5:**
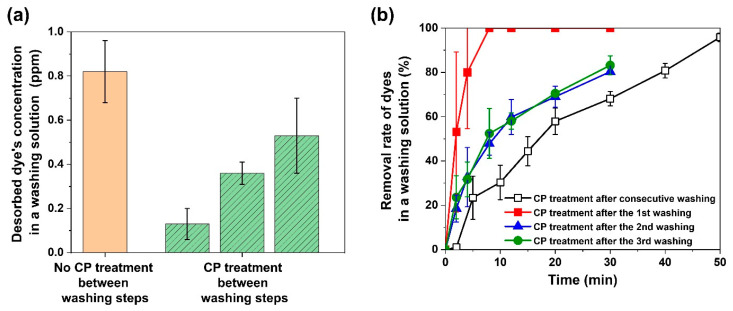
(**a**) Desorbed dye’s concentration in a washing solution measured just before CP treatment began (*n* = 3). (**b**) Removal rate of dyes in a washing solution observed at different times during CP treatment (*n* = 3). The open square indicates the dye removal rate measured during CP treatment after consecutive washing three times without CP treatment between washing steps, while the close square, triangle, and circle signify the cases for the CP treatment between washing steps.

To clarify what brought about the difference in the dye removal efficiencies depending on the used washing solution’s oxidation by CP treatment, we zeroed in on the dye concentrations measured just before CP treatment began. As for the case of three times recycling without CP treatment, the desorbed dye’s concentration was about 0.82 ppm after recycling experiments were conducted in triplicate (Figure 5a). By contrast, when CP treatment was performed between recycling the used washing solution, the desorbed dye’s concentration in the used washing solution showed almost half level (0.53 ppm) in triplicate (Figure 5a). This result signifies that the desorbed dyes in the used washing solution were adequately removed by CP treatment between recycling, successfully regenerating the used washing solution. This interpretation was also evidenced by the trend in the dye removal efficiencies observed at different times. CP treatment eliminated about 80% more than that of the desorbed dyes in 30 min in each recycle (Figure 5b), providing the next washing step with a regenerated cleaning solution of a more favorable condition for adsorbed dyes’ elution and used membranes’ regeneration from the perspective of maintaining membranes’ dye removal performances.

More intriguingly, the CP treatment of used cleaning solution between washing steps was also expected to be beneficial from the perspective of removal kinetics. According to our kinetic study (Table 2), as the used washing solution containing accumulated desorbed dyes was oxidized by CP treatment after three times recycling, the removal kinetic constant was around 0.0318. In stark contrast to this, the removal kinetic constant skyrocketed by equal to or more than 3.7-fold along with the periodic CP treatment between washing steps. This huge difference in the removal kinetic constant is thought to arise from the distinction in the initial dye concentration. In other words, the periodic CP treatment between washing steps is highly likely to lessen the burden imposed by accumulated desorbed dyes by removing the dyes released from every washing step ahead of their accumulation, giving advantages from the perspective of removal kinetics. Furthermore, the significantly higher kinetic constant also suggests that the washing solution’s periodic CP treatment could enable us to considerably reduce the washing solution’s regeneration time in a real process. On the contrary, no periodic CP treatment should end up with a steep increase in the desorbed dye’s accumulation at a larger scale in the long-term process.

However, CP treatment might do more harm than good if it requires energy consumption too heavily, although it can lighten the burden associated with the accumulation of dyes while recycling a used washing solution. For this reason, we evaluated the CP treatment efficiency and energy consumption in the treatment of about half of the desorbed dyes consistently for all CP treatments for a fair comparison. According to our energy consumption analysis (Table 3), it was verified that CP treatment required 15 min and 20.3 kWh m^−3^ order^−1^ to remove about half of the desorbed dyes when the reused washing solution did not go through CP treatment between washing steps. On the other hand, CP treatment between washing steps cut down on the CP treatment time (8 min) and the resulting EEO (~10 kWh m^−3^ order^−1^) to the half level of the case without CP treatment between washing steps, demonstrating that the efficiency of CP treatment was improved by the intermittent CP treatment between washing steps. Thanks to the improved CP treatment efficiency, even the sum (23.3 kWh m^−3^ order^−1^) of the EEO of three times CP treatments between washing steps was similar to the EEO (20.3 kWh m^−3^ order^−1^) for one-time CP treatment after triplicate washing without the intermittent CP treatment. Overall, the intermittent CP treatment between washing steps successfully regenerated the used membrane and washing solution without causing much additional energy consumption.

**Figure 6:**
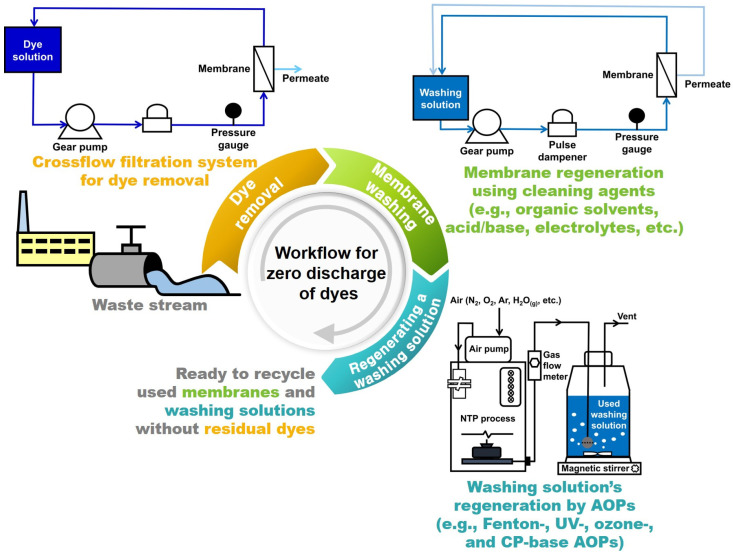
Conceptual illustration of the workflow for zero discharge of dyes in a membrane-based dye removal process via membrane regeneration using various kinds of cleaning agents, such as organic solvents, acid/base, and electrolytes, along with washing solution’s regeneration by diverse AOPs, such as Fenton-, UV-, ozone-, and CP-based AOPs.

Lastly, we discuss the perspectives for the guide to facilitate future work in terms of zero discharge of dyes and regeneration of a washing solution by AOPs, including CP treatment, at a very early stage. First, if a washing solution contains organic compounds, such as alcohol or acetone, one needs to balance between the removals of dyes and organic compound-based cleaning agents by AOPs before they try to regenerate a used washing solution. Accordingly, it is worth carrying out a long-term mechanistic study to investigate the effect of the washing solution’s AOP time and intensity on the regeneration of used membranes and cleaning solutions. Second, electrolyte- or acid/base-based washing solutions are free from concerns about the competitive removal by AOPs. It is extremely rare to find research on the AOP of dyes in inorganic washing agents, such that many things, such as the removal efficiency, remain unanswered. Therefore, there is much room for further study of the AOP of dyes in various kinds of cleaning agents (Figure 6).

## 4. Conclusions

This study demonstrated that cold plasma (CP) treatment worked for the regeneration of membranes and washing solutions used in membrane-based dye removal. When a cleaning solution was repeatedly used with desorbed dyes to wash a used membrane for dye removal, it aggravated the used membrane’s dye removal efficiency from 78.8% to 73.5% in triplicate. This significant reduction in the dye removal efficiency stems from the fact that the loss of adsorption sites occupied by undetached dyes became pronounced by the accumulated dyes when a washing solution was repeatedly used without CP treatment. On the other hand, whenever a used washing solution was treated with CP treatment, most of the desorbed dyes in a washing solution were degraded quickly, regenerating the used washing solution successfully. Owing to the well-regenerated washing solution, the reused membrane’s dye removal efficiency was well-maintained, with only a marginal reduction (78.9% to 75.9%) in triplicate, in contrast to the membrane recycled without CP treatment (78.8% to 73.5%).

## Figures and Tables

**Table 1:** Brief summary of the experimental conditions of the membrane fabrication and filtration and the pure water flux of the UF membrane.

Pressure (Bar)	1.5
Crossflow rate (L min^−^^1^)	1.8–2.1 L min^−1^
Polymer (wt%)	Polysulfone (20 wt%)
Solvent	NMP
Effective membrane area (cm^2^)	13.75
Pure water flux (L m^−2^ h^−1^, *n* = 18)	46.9 (±1.6)

**Table 2:** Kinetic equations of methylene blue removal efficiencies.

CP Treatment Condition	CP Treatment Frequency	Methylene Blue		
		Equation	Kinetic Constant (k, min^−1^)	r^2^
**No CP treatment between washing steps**	CP treatment after triplicate washing	y = 116.4 (1 − e^−0.0318x^)	0.0318	0.9869
**CP treatment between washing steps**	1st CP treatment	y = 101.2 (1 − e^−0.3878x^)	0.3878	0.9981
	2nd CP treatment	y = 80.1 (1 − e^−0.1176x^)	0.1176	0.9933
	3rd CP treatment	y = 80.9 (1 − e^−0.1231x^)	0.1231	0.9822

**Table 3:** Energy consumption of CP treatment during the removal of about half of the desorbed dyes.

CP Treatment Condition	CP Treatment Frequency	Methylene Blue		
		Removal Rate (%)	Treatment Time (min)	EEO (kWh m^−3^ order^−1^)
**No CP treatment between washing steps**	CP treatment after triplicate washing	44.4	15	20.3
**CP treatment between washing steps**	1st CP treatment	53.1	2	3.4
	2nd CP treatment	47.8	8	10.2
	3rd CP treatment	52.4	8	9.7

## Data Availability

Not applicable.

## References

[1] Service R.F. (2006). Desalination freshens up. Science.

[2] Lim Y.J., Lee S.M., Wang R., Lee J. (2021). Emerging materials to prepare mixed matrix membranes for pollutant removal in water. Membranes.

[3] Kim T.-K., Kim T., Lee I., Choi K., Zoh K.-D. (2021). Removal of tetramethylammonium hydroxide (TMAH) in semiconductor wastewater using the nano-ozone H_2_O_2_ process. J. Hazard. Mater..

[4] Li C., Ma H., Venkateswaran S., Hsiao B.S. (2020). Highly efficient and sustainable carboxylated cellulose filters for removal of cationic dyes/heavy metals ions. Chem. Eng. J..

[5] Zereshki S., Daraei P., Shokri A. (2018). Application of edible paraffin oil for cationic dye removal from water using emulsion liquid membrane. J. Hazard. Mater..

[6] Pavithra K.G., Jaikumar V. (2019). Removal of colorants from wastewater: A review on sources and treatment strategies. J. Ind. Eng. Chem..

[7] Selvaraj V., Karthika T.S., Mansiya C., Alagar M. (2021). An over review on recently developed techniques, mechanisms and intermediate involved in the advanced azo dye degradation for industrial applications. J. Mol. Struct..

[8] Radoor S., Karayil J., Jayakumar A., Nandi D., Parameswaranpillai J., Lee J., Shivanna J.M., Nithya R., Siengchin S. (2022). Adsorption of Cationic Dye onto ZSM-5 Zeolite-Based Bio Membrane: Characterizations, Kinetics and Adsorption Isotherm. J. Polym. Environ..

[9] Nie L., Goh K., Wang Y., Lee J., Huang Y., Karahan H.E., Zhou K., Guiver M.D., Bae T.-H. (2020). Realizing small-flake graphene oxide membranes for ultrafast size-dependent organic solvent nanofiltration. Sci. Adv..

[10] Chandarana H., Kumar P.S., Seenuvasan M., Kumar M.A. (2021). Kinetics, equilibrium and thermodynamic investigations of methylene blue dye removal using Casuarina equisetifolia pines. Chemosphere.

[11] Kumar S., Kaushik R., Purohit L. (2021). Hetro-nanostructured Se-ZnO sustained with RGO nanosheets for enhanced photocatalytic degradation of p-Chlorophenol, p-Nitrophenol and Methylene blue. Sep. Purif. Technol..

[12] Bangari R.S., Yadav A., Bharadwaj J., Sinha N. (2022). Boron nitride nanosheets incorporated polyvinylidene fluoride mixed matrix membranes for removal of methylene blue from aqueous stream. J. Environ. Chem. Eng..

[13] Dou T., Zang L., Zhang Y., Sun Z., Sun L., Wang C. (2019). Hybrid g-C3N4 nanosheet/carbon paper membranes for the photocatalytic degradation of methylene blue. Mater. Lett..

[14] Hashem A.H., Saied E., Hasanin M.S. (2020). Green and ecofriendly bio-removal of methylene blue dye from aqueous solution using biologically activated banana peel waste. Sustain. Chem. Pharm..

[15] Sivakumar R., Lee N.Y. (2022). Adsorptive removal of organic pollutant methylene blue using polysaccharide-based composite hydrogels. Chemosphere.

[16] Moradihamedani P. (2021). Recent advances in dye removal from wastewater by membrane technology: A review. Polym. Bull..

[17] Le-Clech P. (2010). Membrane bioreactors and their uses in wastewater treatments. Appl. Microbiol. Biotechnol..

[18] Kraume M., Drews A. (2010). Membrane bioreactors in waste water treatment–Status and trends. Chem. Eng. Technol..

[19] Judd S. (2008). The status of membrane bioreactor technology. Trends Biotechnol..

[20] Lee J., Chae H.-R., Won Y.J., Lee K., Lee C.-H., Lee H.H., Kim I.-C., Lee J.-M. (2013). Graphene Oxide Nanoplatelets Composite Membrane with Hydrophilic and Antifouling Properties for Wastewater Treatment. J. Membr. Sci..

[21] Lee J., Won Y.-J., Choi D.-C., Lee S., Park P.-K., Choo K.-H., Oh H.-S., Lee C.-H. (2019). Micro-patterned membranes with enzymatic quorum quenching activity to control biofouling in an MBR for wastewater treatment. J. Membr. Sci..

[22] Sethunga G., Lee J., Wang R., Bae T.-H. (2019). Influence of membrane characteristics and operating parameters on transport properties of dissolved methane in a hollow fiber membrane contactor for biogas recovery from anaerobic effluents. J. Membr. Sci..

[23] Mehmood C.T., Zhong Z., Zhou H., Xiao Y. (2020). Constructing porous beads with modified polysulfone-alginate and TiO_2_ as a robust and recyclable photocatalyst for wastewater treatment. J. Water Process Eng..

[24] Rouquié C., Szymczyk A., Rabiller-Baudry M., Roberge H., Abellan P., Riaublanc A., Frappart M., Álvarez-Blanco S., Couallier E. (2022). NaCl precleaning of microfiltration membranes fouled with oil-in-water emulsions: Impact on fouling dislodgment. Sep. Purif. Technol..

[25] Xu Y., Li Z., Su K., Fan T., Cao L. (2018). Mussel-inspired modification of PPS membrane to separate and remove the dyes from the wastewater. Chem. Eng. J..

[26] Yan J., Huang Y., Miao Y.-E., Tjiu W.W., Liu T. (2015). Polydopamine-coated electrospun poly (vinyl alcohol)/poly (acrylic acid) membranes as efficient dye adsorbent with good recyclability. J. Hazard. Mater..

[27] Liu C., Cheng L., Zhao Y., Zhu L. (2017). Interfacially crosslinked composite porous membranes for ultrafast removal of anionic dyes from water through permeating adsorption. J. Hazard. Mater..

[28] Yang F., Sadam H., Zhang Y., Xia J., Yang X., Long J., Li S., Shao L. (2020). A de novo sacrificial-MOF strategy to construct enhanced-flux nanofiltration membranes for efficient dye removal. Chem. Eng. Sci..

[29] Kim H.-J., Won C.-H., Kim H.-W. (2020). Optimized Pretreatment of Non-Thermal Plasma for Advanced Sewage Oxidation. Int. J. Environ. Res. Public Health.

[30] Lv J., Zhao F., Feng J., Liu Q., Nan F., Liu X., Xie S. (2020). The impact of particulate and soluble organic matter on physicochemical properties of extracellular polymeric substances in a microalga Neocystis mucosa SX. Algal Res..

[31] Kim H.-J., Won C.-H., Hong Y.-P., Lee I.H., Kim H.-W. (2021). Energy-effective elimination of harmful microcystins by a non-thermal plasma process. Chemosphere.

[32] Zeghioud H., Nguyen-Tri P., Khezami L., Amrane A., Assadi A.A. (2020). Review on discharge Plasma for water treatment: Mechanism, reactor geometries, active species and combined processes. J. Water Process Eng..

[33] Kwak D.-H., Jee S.-I., Kim H.-J., Won C.-H. (2018). Feasibility of glow discharge nonthermal plasma as an alternative pretreatment for low-carbon wastewater in a biological nutrient removal plant. Environ. Eng. Sci..

[34] Park R., Kim J.-G., Kim H.-W. (2021). Prediction of varying microcystins during non-thermal plasma oxidation of harvested microalgal biomass. J. Hazard. Mater..

[35] Wang B., Dong B., Xu M., Chi C., Wang C. (2017). Degradation of methylene blue using double-chamber dielectric barrier discharge reactor under different carrier gases. Chem. Eng. Sci..

[36] Wu L., Xie Q., Lv Y., Wu Z., Liang X., Lu M., Nie Y. (2019). Degradation of methylene blue via dielectric barrier discharge plasma treatment. Water.

[37] Lee J., Jang J.H., Chae H.-R., Lee S.H., Lee C.-H., Park P.-K., Won Y.-J., Kim I.-C. (2015). A facile route to enhance the water flux of a thin-film composite reverse osmosis membrane: Incorporating thickness-controlled graphene oxide into a highly porous support layer. J. Mater. Chem. A.

[38] Kim H.-J., Nam G.-S., Jang J.-S., Won C.-H., Kim H.-W. (2019). Cold plasma treatment for efficient control over algal bloom products in surface water. Water.

[39] Park J.-A., Yang B., Park C., Choi J.-W., van Genuchten C.M., Lee S.-H. (2017). Oxidation of microcystin-LR by the Fenton process: Kinetics, degradation intermediates, water quality and toxicity assessment. Chem. Eng. J..

[40] Misdan N., Lau W., Ismail A., Matsuura T. (2013). Formation of thin film composite nanofiltration membrane: Effect of polysulfone substrate characteristics. Desalination.

[41] Schwarze M., Schaefer L., Chiappisi L., Gradzielski M. (2018). Micellar enhanced ultrafiltration (MEUF) of methylene blue with carboxylate surfactants. Sep. Purif. Technol..

[42] Khumalo N.P., Vilakati G.D., Mhlanga S.D., Kuvarega A.T., Mamba B.B., Li J., Dlamini D.S. (2019). Dual-functional ultrafiltration nano-enabled PSf/PVA membrane for the removal of Congo red dye. J. Water Process Eng..

[43] Kadhim R.J., Al-Ani F.H., Al-Shaeli M., Alsalhy Q.F., Figoli A. (2020). Removal of dyes using graphene oxide (GO) mixed matrix membranes. Membranes.

[44] Safarpour M., Najjarizad-Peyvasti S., Khataee A., Karimi A. (2022). Polyethersulfone ultrafiltration membranes incorporated with CeO2/GO nanocomposite for enhanced fouling resistance and dye separation. J. Environ. Chem. Eng..

[45] Salimi A., Roosta A. (2019). Experimental solubility and thermodynamic aspects of methylene blue in different solvents. Thermochim. Acta.

